# COVID‐19 vaccine hesitancy of older people in China

**DOI:** 10.1002/ctm2.1397

**Published:** 2023-09-10

**Authors:** Gewei Wang, Yao Yao, Yafeng Wang, Jinquan Gong, Qinqin Meng, Hui Wang, Wenjin Wang, Xinxin Chen, Yaohui Zhao

**Affiliations:** ^1^ Institute of Social Science Survey Peking University Beijing China; ^2^ China Center for Health Development Studies Peking University Beijing China; ^3^ China Center for Economic Research National School of Development Peking University Beijing Beijing China; ^4^ Institute for Global Health and Development Peking University Beijing Beijing China

The COVID‐19 pandemic has underscored the pivotal role of vaccination in averting life‐threatening illnesses among older adults. Despite the rollout of domestically manufactured COVID‐19 vaccines and their free distribution to the general public, many older individuals in China still hesitate to receive vaccination. To enhance vaccine acceptance and uptake, it is crucial to understand and address the concerns of vaccine‐hesitant individuals, particularly older adults with underlying health conditions. In a recent publication, 'Determinants of COVID‐19 Vaccination Status and Hesitancy Among Older Adults in China', we examined the factors underlying vaccine hesitancy among older adults in China and provided suggestions for future vaccine campaigns.^1^

## 1 VACCINE HESITANCY AMONG CHINESE OLDER PEOPLE

Chinese‐made vaccines, including Sinopharm and Sinovac, were approved for nationwide vaccination in January 2021, but older adults were initially excluded due to the lack of clinical trial data. Although vaccination for older adults became available in June 2021, many still did not complete their vaccinations. In addition, vaccinators refused vaccinations for some older people with underlying health conditions due to the lack of clear definitions of COVID‐19 vaccine contraindications. The government emphasised full vaccination of older people, especially the oldest old, after ending the 'zero‐COVID' policy in November 2022. However, massive infections sweeping the country in December 2022 and January 2023 made it impossible to implement. Understanding specific influencing factors, such as the focus and motivation that affect older people's vaccination decisions, can provide lessons for future vaccination campaigns. A fifth nationally representative survey conducted by CHARLS between July 2021 and August 2022 included related questions about COVID‐19 vaccination status, reasons for non‐vaccination and influencing factors to address the issue of vaccine hesitancy among older Chinese people. The survey data from the nationally representative sample of older people reflected their vaccination status when the 'zero COVID' policy ended in China as the vaccination rate among China's older population changed little from August to November 2022.

As of July–August 2022, 12 900 participants with a mean age of 65.2 years were included in the vaccine status analysis. The weighted vaccination rate (at least one dose) for Chinese older people aged 60 and above was 92.3%; while the full course and booster shot vaccination rates for this group were 88.6% and 72.4%, respectively. The vaccination rate decreases with age. In the oldest‐old group (80 and above), the proportion of people who received one dose was 80.5%; however, the proportion of this population who received the full course and booster shots was only 71.9% and 46.7%, respectively.

## 2 FACTORS THAT INFLUENCE VACCINE UPTAKE

Although the government's promotion of vaccine popularisation activities from July 2021 to August 2022 significantly increased the vaccination rate among vulnerable groups such as the older, ethnic minorities, and people with disabilities, a considerable proportion of the population is still not covered by vaccination.

In the study, we found that older individuals (particularly those aged 80 years and above), women, unmarried persons, urban residents, individuals with disabilities, and those with chronic conditions (such as hypertension, heart disease, stroke, diabetes, lung disease, and cancer) were less likely to receive COVID‐19 vaccination. A comparison of vaccination status during the summers of 2021 and 2022 revealed that during the first phase (within 6 months of the introduction of domestic COVID‐19 vaccines in 2020), the probability of vaccination was lower among women, older individuals (especially those aged 80 and above), unmarried and widowed individuals, non‐Han ethnic groups, urban residents, those with functional disabilities, and those with chronic diseases. During the second phase (between the summers of 2021 and 2022), the probability of vaccination was significantly higher among high‐risk populations, particularly the older ones, individuals with functional disabilities, and those with major chronic conditions (which were often erroneously perceived as contraindications to the COVID‐19 vaccine), reflecting the government's endeavours and accomplishments in reducing vaccine uptake disparities.

The self‐reported reasons for not receiving COVID‐19 vaccination in our study corroborated our analysis results. The top five reasons reported by participants between July and August 2022 were concerns about contraindications (48%), old age/weakness/chronic illness (21%), accessibility issues due to mobility or supply problems (18%), concerns about side effects or efficacy (9%), and lack of awareness of the COVID‐19 vaccine (6%). Notably, lack of awareness of the vaccine was more common among older individuals, while concerns about contraindications were more prevalent among younger older people. Although the proportion of accessibility issues and lack of awareness of the vaccine has decreased since the summer of 2021, the proportion of concerns about contraindications has increased over the past year, from 30% to 48% in the summer of 2022, despite the government's efforts to promote vaccine coverage activities.

## 3 STRATEGIES TO INCREASE VACCINE UPTAKE

China's experience offers significant lessons for the global pandemic response, specifically in achieving an effective balance between controlling viral spread through measures like quarantine and reducing the negative impacts of COVID‐19 through vaccination and treatment. During the initial outbreak, control measures can significantly prevent virus spread without vaccines. Once vaccines are available, efforts should focus on vaccinating vulnerable populations and improving treatment capacity (such as building ICUs). As vaccine coverage is promoted, a clear and gradual timetable should be set for lifting control measures, ensuring the public is well‐informed and can plan vaccination arrangements accordingly. Our study revealed that among the older population in China, vaccine hesitancy mainly results from misunderstandings about contraindications to COVID‐19 vaccines and excessive concerns about vaccine side effects. Therefore, before initiating vaccine promotion campaigns, it is necessary to build public trust by clarifying vaccine side effect issues and scientifically explaining the contraindications related to COVID‐19 vaccines. In the meantime, healthcare providers and physicians remain the most trusted sources of vaccination advice. Therefore, healthcare providers must support, encourage patients and listen to their concerns. Providing physicians with knowledge of the specific concerns within their communities can aid them in addressing these concerns in the clinic and formulating effective interventions at the community level.^2^ It is worth noting that vaccine hesitancy and skepticism may be exacerbated by the pervasive influence of social media platforms, where the 'anti‐vaccine' message is more unregulated than in other forms of media.^3^ Strengthening digital monitoring and regulation and addressing stigmatisation surrounding COVID‐19 vaccines could aid in preventing uninformed vaccine decisions by the public.
